# Demographic, clinical and behavioural determinants of HIV serostatus non-disclosure to sex partners among HIV-infected pregnant women in the Eastern Cape, South Africa

**DOI:** 10.1371/journal.pone.0181730

**Published:** 2017-08-24

**Authors:** Oladele Vincent Adeniyi, Anthony Idowu Ajayi, Nonkosi Selanto-Chairman, Daniel Ter Goon, Gerry Boon, Yusimi Ordaz Fuentes, George Justus Hofmeyr, Gordana Avramovic, Craig Carty, John Lambert, on behalf of the East London Prospective Cohort Study (ELPCS) Group

**Affiliations:** 1 Department of Family Medicine and Rural Health, Walter Sisulu University, Cecilia Makiwane Hospital/East London Hospital Complex, East London, South Africa; 2 Department of Sociology, University of Fort Hare, East London, South Africa; 3 Buffalo City Metro Department of Health, East London, South Africa; 4 Faculty of Health Sciences, University of Fort Hare, East London, South Africa; 5 Department of Paediatrics, Walter Sisulu University, Frere Hospital/East London Hospital Complex, East London, South Africa; 6 Department of Family Medicine, Cecilia Makiwane Hospital/East London Hospital Complex, East London, South Africa; 7 Effective Care Research Unit, Walter Sisulu University, Frere hospital/East London Hospital Complex, East London, South Africa; 8 University College of Dublin/Mater Misericordiae University Hospital, Catherine McAuley Education & Research Centre, Dublin, Ireland; 9 The Relevance Network, Johannesburg, South Africa; 10 University College Dublin/Mater Misericordiae University Hospital, Catherine McAuley Education & Research Centre, Dublin, Ireland; China Medical University, CHINA

## Abstract

**Objectives:**

Drawing from a baseline sample of a cohort study, the study examines the extent and correlates of serostatus non-disclosure to sex partners and family members, and reasons for non-disclosure among HIV-infected pregnant women in the Eastern Cape Province, South Africa.

**Methods:**

This longitudinal cohort study recruited 1709 pregnant women living with HIV who attended three of the largest maternity centres in the Eastern Cape, South Africa, for delivery between September 2015 and May 2016. Relevant items on demographics, serostatus awareness, disclosure to sex partners and family members, and lifestyle behaviours were obtained using structured interviews. Age-stratified binary logistic regression models were used to determine the significant correlates of non-disclosure among the participants.

**Results:**

A higher rate of HIV serostatus non-disclosure to sex partners (25.6%) in comparison to family members (20%) was reported by the participants. Younger age, not living with partners and alcohol use were significantly associated with non-disclosure of HIV serostatus to sex partners. Non-disclosure of HIV serostatus to sex partners was significantly (p<0.05) associated with poor adherence to the highly active anti-retroviral therapy (HAART), failure to keep clinic appointments and high viral load at the delivery of the baby. Perceived fear of intimate partner violence, fear of rejection, guilt of not disclosing at the onset of the relationship, sex partner’s non-disclosure of HIV serostatus, and guilt of unfaithfulness were some of the reasons for non-disclosure of HIV serostatus to sex partners.

**Conclusions:**

Non-disclosure of HIV serostatus is a public health concern with serious implications for both mother-to-child transmission, as well as horizontal transmission, in our setting. Strategic efforts toward ending the epidemic of HIV and AIDS in South Africa should address the sociocultural and behavioural determinants of non-disclosure.

## Introduction

The Sustainable Development Goal 3.3 aims to end the epidemics of AIDS by the end of year 2030. In South Africa, a country with one of the highest burdens of HIV globally, about seven million individuals are living with HIV [1]. The country is among the few that have demonstrated much commitment towards ending the AIDS epidemic. Paramount to eliminating the AIDS epidemic are prevention of new infections and provision of treatment for people living with HIV. South Africa is doing well in increasing access to the highly active antiretroviral therapy. However, considerable gaps still exist in strategies towards preventing new infections. Disclosure of HIV serostatus to partners could play a role in facilitating couple testing and home testing that are part of the strategies being considered towards achieving an HIV-free generation.

The review of the literature shows that there is a wide variation in the proportion of HIV serostatus disclosure to sex partners reported between populations and across different study contexts [2–5]. Thus, the need for context-specific data to drive interventions becomes crucial. This is lacking in the Eastern Cape, an understudied region in South Africa.

The link between non-disclosure of HIV serostatus to sex partners and transmission of HIV infection is well documented [6–8]. Also, researchers have explored the link between non-disclosure of HIV serostatus and non-adherence to medications [9, 10]. In contrast, serostatus disclosure to sex partners and/or to family members has been demonstrated to be beneficial [11, 12]. HIV serostatus disclosure has been shown to facilitate social support; self-acceptance of serostatus; improve psychological well-being among HIV-infected individuals; increase sexual communication; promote HIV testing and HIV prevention among sex partners [13–15]. Trinh [16] asserts that HIV serostatus disclosure to sex partners is associated with CD4 recovery following antiretroviral therapy (ART) initiation.

Despite the benefits associated with HIV serostatus disclosure, studies have shown that this is not always straightforward due to fear of partner violence; fear of stigma and discrimination; fear of abandonment and accusation of infidelity [5, 17–21]. Nevertheless, studies on outcomes of HIV serostatus disclosure to sex partners showed that the majority of women that had disclosed their status were accepted, received emotional and financial support and they had the freedom to use the highly active anti-retroviral therapy (HAART). Only a very few of them experienced physical abuse and blame [19, 20, 22].

As highlighted above, disclosure of HIV serostatus is beneficial and non-disclosure could have deleterious consequences. In view of this, many researchers are focusing attention on factors associated with serostatus (non-)/disclosure. A number of factors identified to be associated with disclosure include; having attended a HIV counselling programme or belonging to a support group, duration of knowledge of HIV status, gender, age, whether the partnership is regular rather than casual or an unfamiliar relationship, whether the person being disclosed to has a known positive status, perceived less stigma, having a partner with tertiary education, less financial dependence on partners, less experience of violence and knowing someone with HIV [14, 16, 23–25].

A few studies have linked non-disclosure of HIV serostatus with sub-optimal prevention of mother-to-child transmission (PMTCT) (late initiation of antiretroviral therapy, detectable viral load at delivery and lack of neonatal prophylaxis) [16, 26]. However, the evidence of this link is less certain and thus, indicates the need for further study in light of the anticipated policy on couple testing and optimisation of the PMTCT programme in South Africa. This study adds to existing knowledge by using data from a large cohort study to determine the prevalence and correlates of serostatus non-disclosure to sex partners and family members in a cohort of pregnant women living with HIV in the Eastern Cape, South Africa.

## Methods

### Study design and settings

The data analysed in the study came from a baseline sample of the East London Prospective Cohort Study, which was conducted, between September 2015 and May 2016 across three large maternity facilities in the Buffalo/Amathole districts in the Eastern Cape Province, South Africa. These health facilities serve a combined population of 1,674,637 people with Amathole district slightly more populated (892,637) than Buffalo City metropolitan (755,000) [27]. HIV prevalence at population level is 12.7% while the prevalence among pregnant women in the region is 30% [27]. The selected health facilities represent the various demographics and levels of health care in the province. Frere hospital is an urban tertiary health facility which receives referrals of patients across the region, while Cecilia Makiwane hospital, a regional health facility, is located in the semi-urban Mdantsane township and provides both level one and two services in the region. Bisho hospital is a district health facility serving a predominantly rural population of Bisho and its surrounding communities.

In 2015, the South African government implemented the WHO Option B+ strategy (life-long HAART) towards achieving the goal of elimination of mother-to-child transmission (MTCT) of HIV. The field performance of this evidence-based strategy might inform the health managers on the effectiveness of the implementation across health facilities. Hence, the East London Prospective Study was conducted to generate robust epidemiological data for guiding policies on PMTCT in the region. All pregnant women are offered provider-initiated counselling and testing at booking and throughout pregnancy, delivery and at immunization clinics. Pregnant women diagnosed with HIV are provided adherence counselling and HAART, in accordance with the PMTCT guideline [28]. Numerous antenatal clinics, however, do not provide delivery services and thus, pregnant women access maternity services at the hospitals and some community health centres in their communities.

### Participants and sample size

The sample size of the East London Prospective Cohort Study was based on the estimated proportion of HIV-infected parturient retained in care after one year post-delivery in the study population [29]. We estimated a sample size of 1709 participants after adjusting for a probable 10% loss to follow-up within the first six months post-delivery and allowing for a confidence interval of 2.5% with a confidence level of 95%. Since the study participants were recruited from three large clinics that serve the Buffalo/Amathole districts in the Eastern Cape Province, South Africa, our sample size is representative of these two districts in the Eastern Cape Province. It is highly likely that our findings could possibly be representative of the entire Eastern Cape province.

All HIV-infected pregnant women who attended the maternity centres of the selected hospitals during the study period were eligible to participate. There was no refusal among the parturient.

All participants underwent the standard of care in accordance with the recommendations of the South African Department of Health (NDOH, 2015). Participants were recruited serially at the post-natal wards of maternity centres within 24 hours of vaginal delivery and 72 hours for caesarean section delivery. Trained research assistants conducted face-to-face interviews using structured questionnaires. Research nurses checked the viral load of the participants and drew venous blood in patients who had no documented viral load within the month of delivery.

An electronic datasheet was designed and piloted with ten pregnant women at one of the study centres to ascertain the validity of the instrument and research process. Adjustments were made using feedback from the participants and the research team.

### Measures

#### Socio-demographic variables

Participants provided information about their age, level of education, marital status and place of residence. Participants were asked about their lifestyle behaviours; cigarette smoking status before and during the index pregnancy as well as their alcohol consumption.

#### Clinical variables

The number of deliveries (parity), gestational age at booking, HIV serostatus at booking and whether they were already on HAART, contraceptive choices, adherence to HAART, recent viral load and opportunistic infections in the index pregnancy were extracted from the clinical records of each participant.

#### Non-disclosure of HIV serostatus

Non-disclosure of HIV serostatus was assessed by measures created for the study. Participants were asked whether they had disclosed their serostatus to their sex partners (associated with the index pregnancy): (yes/no). If the answer was “No”, the concerns and worries related to disclosure to the sex partner were elicited through the question, “can you elaborate on the reason(s) for not disclosing your status? Participants also provided answers as to whether they had disclosed their status to a family member and they elaborated on the relationship with the family member.

#### Impact of non-disclosure

Specific measures evaluating the impact of non-disclosure of HIV status were assessed. Participants were asked about their adherence to their ARVs in the week prior to the interview and prior defaulting on use of ARVs. Also, scheduled appointments of each participant and viral load at delivery were extracted from their medical records.

### Ethical consideration

Ethical approval was obtained in line with the standard procedures from the Walter Sisulu University Ethics Committee and the Eastern Cape Department of Health. The management of the respective hospitals gave permission for the implementation of the study protocol. Participants were provided with an information sheet written in English and a translated version in IsiXhosa, detailing the purpose and the process of the study. Each participant gave written, informed consent for voluntary participation in the study. A few participants under the age of 18 years were assisted by their legal guardians, while they gave assent for their involvement in the study. Participants’ rights to privacy and confidentiality were respected throughout the study period.

## Data analysis

Descriptive statistics (means, proportions and standard deviations) were used to describe the socio-demographic characteristics of the participants and disclosure to sex partners and family members. Non-disclosure of HIV status to sex partners and family members was the primary outcome of this analysis. The significant associations between outcome variable (HIV disclosure to sex partners and family members) and explanatory variables were examined by carrying out a bivariate analysis using the chi square test. Bivariate logistic regression models tested independent associations between variables and level of significance (α = 0.05). Data were analysed using Statistical Package for Social Sciences version 21.0 (SPSS, Chicago, IL, USA).

## Results

### Socio-demographic characteristics

A total of 1709 pregnant women living with HIV participated in the study and their age ranged from 14 to 47 years with a mean (±SD) of 29.63 (±6.2) years. The majority of the study participants were single (69.5%), unemployed (74.7%), had grade 12 education (86.5%), and two or more children (60.5%). Table 1 presents the socio-demographic characteristics of participants.

**Table 1:** Demographic characteristics.

Variable	Frequency (1709)	Percentage
**Age**		
≤19	60	3.5
20–24	331	19.5
25–29	459	27.0
30–34	452	26.6
35–39	303	17.8
40–44	96	5.6
**Marital Status**		
Married	312	18.3
Single	1187	69.5
Cohabiting	186	10.9
Divorce/Separated	24	1.4
**Place of residence**		
Rural	585	34.2
Semi urban	792	46.3
Urban	332	19.4
**Education Level**		
No formal Education	5	0.3
Grade 1–6	115	6.7
Grade 7–12	1479	86.5
Tertiary	110	6.4
**Employment Status**		
Unemployed	1277	74.7
Employed	432	25.3
**Alcohol Use**		
Drank during pregnancy	230	13.8
Quit drinking during pregnancy	431	25.2
Never drank	1043	61.0
**Smoking status**		
Smoked during pregnancy	92	5.9
Quit smoking during pregnancy	80	4.7
Never smoked	1529	89.5
**Gestational age at booking**		
First trimester	210	12.3
Second	1229	71.9
Third	270	15.8
**Knowledge of HIV status prior to booking**		
Positive	1356	80.1
Negative	87	6.7
Unknown	233	13.2
**On HAART at booking**		
No	390	28.1
Yes	998	71.9
**Parity**		
1	521	30.5
≥2	1188	60.5

The majority of the women (80.1%) already knew their HIV status prior to the index pregnancy and of those that knew their status, 71.9% were on HAART at first antenatal care (booking). Of all the participants, 10.6% were smokers, however, half of them quitted smoking during pregnancy. Likewise, about 39% of the participants were alcohol users but the majority of them (65.2%) quitted drinking during pregnancy. All women reported to have received counseling on infant feeding options, on availability of antiretroviral drugs (ARVs) to reduce MTCT of HIV, on contraceptive options, importance of partner disclosure and bringing their partner for HIV counseling and testing.

### Disclosure of HIV serostatus

**Fig 1:**
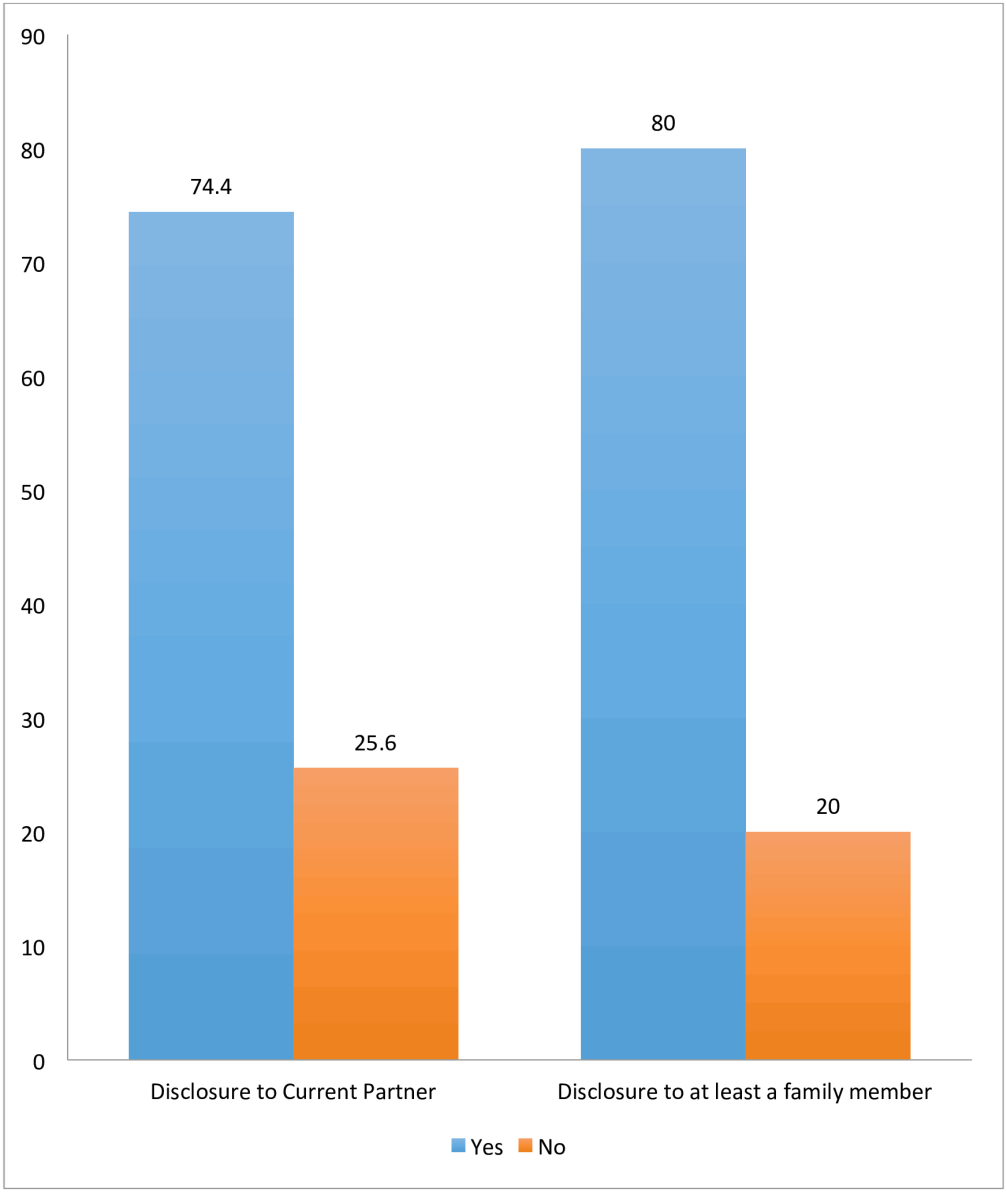
Prevalence of HIV serostatus disclosure to sex partners and at least a family member.

A total of 1253/1684 pregnant women (74.4%) had disclosed their HIV serostatus to their sex partners (Fig 1). A higher proportion of the participants (80.0%) had disclosed their HIV serostatus to at least a family member. Of those who had disclosed their HIV serostatus to a family member; 52.7% disclosed to their mothers, 24.7% to their sisters and 13.3% to an extended family member. Only a few of them disclosed their serostatus to their fathers (2.5%) or brothers (3.3%).

### Correlates of HIV serostatus non-disclosure to sex partners

Table 2 shows the result of bivariate analysis of correlates of HIV non-serostatus disclosure to partners. There was a significant positive correlation between age and the non-disclosure of HIV serostatus to sex partners. The proportion of participants that had not disclosed their HIV serostatus reduced with increase in age. Likewise, not living with sex partners was significantly associated with non-serostatus disclosure. Most women (88%), who were married or cohabiting, had disclosed their status to their sex partners. However, the place of residence was not significantly associated with disclosure of HIV non-serostatus to sex partners.

**Table 2:** Correlates of HIV non-serostatus disclosure to partners.

Variable	No	Yes	p-value
**Age**			
≤24	137(35.6)	248(64.4)	<0.001
≥25	293(22.6)	1001(77.4)	
**Marital Status**			
Not living with partner	372(31.2)	820(68.8)	<0.001
Living with partner	59(12.0)	433(88.0)	
**Place of residence**			
Rural	151(26.1)	427(73.9)	0.376
Semi urban	188(24.2)	590(75.8)	
Urban	92(28.0)	236(72.0)	
**Employment status**			
Unemployed	335(26.7)	922(73.3)	0.049
Employed	96(22.5)	331(77.5)	
**Alcohol Use**			
Alcohol users	212(32.3)	445(67.7)	<0.001
Non-alcohol users	219(21.3)	808(78.7)	
**Smoking habit**			
Smokers	58(33.5)	115(66.5)	0.009
Non-smokers	373(24.7)	1138(75.3)	
**Knowledge of status at booking**			
No	107(33.8)	210(66.2)	<0.001
Yes	323(23.7)	1042(76.3)	
**Disclosure to Family**			
No	136(40.4)	201(59.6)	<0.001
Yes	295(21.9)	1052(78.1)	
**Defaulted antiretroviral**			
Yes	62(31.8)	133(68.2)	0.013
No	335(24.0)	1063(76.0)	
**Self-reporting of non-adherence**			
No	114(30.8)	256(69.2)	0.003
Yes	296(23.4)	968(76.6)	
**Peripartum viral load**			
Not suppressed	82(31.7)	177(68.3)	0.005
Suppressed	283(23.7)	912(76.3)	
**Parity delivery**			
1	153(29.8)	360(70.2)	0.005
≥2	278(23.7)	893(76.3)	

ARV = Antiretroviral.

Women that were employed were slightly more likely to have disclosed their HIV status to their sex partners. Non-users of alcohol, non-smokers and women with prior knowledge of their HIV status were more likely to have disclosed their status to their partners compared to users of alcohol, smokers and women that only knew their status at antenatal care booking.

Women who had disclosed their HIV status to a family member were more likely to disclose their status to their sex partner. Non-disclosure of HIV serostatus was associated with high peripartum viral load (≥1000 RNA copies/ml; mean 64,538 RNA copies/ml; range 1000–2,761,142 RNA copies/ml). Similarly, non-disclosure was associated with self-reported poor adherence to medication and regular pick-up of HAART from the clinics. Also, parity was associated with disclosure of HIV serostatus to partners.

In the logistic regression analysis, after adjusting for confounding variables (employment status, smoking status, parity, place of residence and self-report of non-adherence), women had increased odds of not disclosing their HIV status if; they were less than 25 years old (AOR = 1.6; 95% CI:1.2–2.1), were not living with their partners (AOR = 3.6; 95% CI:2.6–5.0), used alcohol (AOR = 1.7; 95% CI:1.3–2.2), were diagnosed at antenatal care booking (AOR = 1.4; 95% CI:1.1–1.9) and had virological failure at delivery (AOR = 1.4; 95% CI:1.1–1.9) (Table 3).

**Table 3:** Logistic regression predicting serostatus non-disclosure to sex partners.

Variable	AOR	95% CI	p-value
**Age**			
≤24	1.6	1.2–2.1	0.001
≥25			
**Marital Status**			
Not living with partner	3.6	2.6–5.0	<0.001
Living with partner			
**Prior Knowledge of serostatus**			
No	1.4	1.1–1.9	0.021
Yes			
**Use Alcohol**			
Yes	1.7	1.3–2.2	<0.001
No			
**Peripartum viral load suppression**			
No	1.4	1.0–1.9	0.034
Yes			

AOR = adjusted odds ratio, CI = confidence interval.

In the logistic regression analysis, after adjusting for confounding variables (age, smoking status, alcohol use, parity, place of residence and self-report of non-adherence), women had increased odds of not disclosing their HIV status to a family member if; they had not disclosed to their sex partners (AOR = 2.6; 95% CI:1.9–3.5), did not regularly pick up HAART (AOR = 1.5; 95% CI:1.1–1.2.0) and were only aware of their status at antenatal care booking (AOR = 2.6; 95% CI:1.9–3.5). Women that were unemployed (AOR = 0.7; 95% CI:0.5–0.9) and not living with their partner (AOR = 0.5; 95% CI: 0.4–0.7) were less likely to not disclose their HIV status to at least a family member (Table 4).

**Table 4:** Logistic regression predicting serostatus non-disclosure to a family member.

Variable	AOR	95% CI	p-value
**Employment status**			
Unemployed	0.7	0.5–0.9	0.020
Employed			
**Disclosure to Sex Partners**			
No	2.6	1.9–3.5	<0.001
Yes			
**Pick up ARV**			
No	1.5	1.1–2.0	0.020
Yes			
**Marital Status**			
Not living with partner	0.5	0.4–0.7	<0.001
Living with partner			
**Prior Knowledge of status**			
No	2.6	1.9–3.5	<0.001
Yes			

CI = Confidence interval; AOR = adjusted odds ratio; ARV = Antiretroviral.

### Narrative of participants on reasons for non-disclosure of HIV status to their sex partners

Participants’ willingness to disclose their HIV serostatus seems to have depended on the perceived potential reactions of their sex partners. Perceived non-acceptance of their status and non-willingness to accept blame for transmission of HIV to their partners were the dominant reasons why women did not disclose their status to their partners. This view was echoed in the responses of the participants:

“I’m not ready to tell him, I don't trust him that he will accept my status”

*(29 years old para 3)*.

“I am scared to tell him; I think he will blame me. I don't know how I got HIV”

*(35 years old para 3)*.

Disclosure of HIV serostatus also depended on the attitude of the sex partner in question. Partners that were disposed to violence made it almost impossible for women to declare their status because of their fear of a possible violent backlash. The response of a 25-year-old participant below highlights this finding;

“I’m scared to tell him, I don't have the guts to tell him, he is a big bully and rude”

*(25year old para 2)*.

Also, the status of the relationship was a possible determinant of HIV serostatus disclosure. Participants that were no longer in a relationship were most likely never going to disclose to their ex-partners. As highlighted in the response of a 21-year-old participant below, disclosing to an ex-partner could be humiliating and unnecessary;

“*We are not in a relationship anymore so I don't see the need to tell him*. We are not dating anymore”.

The quantitative results indicated that participants that were living with their partners were more likely to disclose their serostatus compared to participants that did not currently live with their partners. This finding was corroborated by the response of a 38-year-old participant below;

“*He is not around*, *he is in Cape Town and he also doesn't have a phone”*.

The aforementioned reasons suggested that taking responsibility for contracting HIV infection could influence disclosure of serostatus; women who believed they were responsible for the transmission of HIV and possibly, had infected their sex partners were more likely not to disclose their status. This finding was corroborated by the responses below;

“I don’t see the need to tell him because I knew that I cheated on him”

*(23 years old para 1)*.

**“**‘I’m scared to tell him now; I didn't want to tell him my status because I didn't tell him that I’m HIV positive when we met”

*(36 years old para 4)*.

Lastly, some participants opined that men were supposed to be the first to disclose their status. This assertion called into question the issue of balance of power in the relationship and how this influenced decision-making. Women who were unable to persuade their partner to test for HIV, or whose partners refuse to inform them of their status, felt they were not obliged to inform their partners. This assertion featured in the views of the participants below;

“I’m not ready, I won't tell him my status until he tells me his”

(29 years old para 2)

“I am not ready to tell him; I think he is also not telling me his HIV status”

*(27years old para 2)*.

## Discussion

The study examined the prevalence and correlates of HIV serostatus disclosure to sex partners, and reasons for non-disclosure in a cohort of parturient living with HIV. The finding of a high rate of HIV serostatus disclosure among the participants is similar to a previous report by Simbayi et al., in their South African study, but higher than Makin et al’s [25] study conducted in Tshwane, South Africa. The prevalence rate found in this study is also higher than what was reported in similar studies in other sub-Saharan Africa countries such as; Uganda [30] and Nigeria [31, 32]. However, higher rates of disclosure to sex partners were reported in Tanzania [22], Malawi [33], Namibia [23], Kenya [23] and Ethiopia [11, 22].

Findings from this study show that HIV serostatus disclosure is associated with older age, being married, having disclosed to a family member and prior knowledge of HIV serostatus before the index pregnancy. Our finding shows that women above the age of 24 years are more likely than younger women to disclose their status, which is consistent with a report by Ahn et al’s [34]. Perhaps, older women disclose their status due to the maturity associated with age or probable longer duration of the relationship. Another plausible explanation could be that older women are more likely to know how to start disclosure communication, assume equal power in the relationship and are of better socioeconomic status. However, this finding is in contrast to Mayfield et al’s [35] study that reported higher prevalence of HIV serostatus disclosure in younger women.

Another important finding of this study is that women currently living with their sex partners are more likely to disclose their status when compared to women who do not. Perhaps, the shared responsibility and high level of commitment associated with marriage or co-habitation suggest that such an important issue would be discussed in such settings. On the other hand, it is possible that living with a sex partner could afford women more opportunity to bring up HIV testing discussions, which might pave way for the subsequent disclosure of HIV serostatus. Sendo et al [36] assert that prior discussion about testing and a smooth relationship are associated with HIV serostatus disclosure to sex partners. Considering that the prevalence of HIV is higher in single women than in married women, non-disclosure of serostatus to sex partners could further lead to horizontal spread of HIV infection. This finding is in contrast to Kiula et al’s [5], but similar to Antelman et al’s [3] study in Dar es Salam and Makin et al’s [25] study in South Africa.

Women who had given birth before are more likely to disclose their status when compared to women who are yet to. The plausible explanation for this is that women who had given birth before are more likely to have known their status long before the current pregnancy, initiated ARVs, accepted their status, attended many counseling sessions and belong to support groups, hence, they are better equipped to disclose their status.

Our findings indicate that disclosure to a family member was higher in comparison to disclosure to sexual partner. Nonetheless, the high proportion of women who had both disclosed their status to their family members and sex partners suggest that both are inter-related.

One interesting finding of this study is that high proportion of the women disclosed their sero-status to female family members, but very few of them disclosed to their male family members. A possible reason for very low disclosure rate to fathers in particular is that most fathers are absent in black South Africa families [37–39].

A surprising finding of this study is the association between alcohol use and disclosure of HIV serostatus. Indeed, the link between alcohol use and risky sexual behaviour had been established by previous studies. Alcohol users who have multiple partners are less likely to disclose their HIV serostatus to their partners and generally, the low rate of disclosure of HIV serostatus to sex partners has been reported among users of alcohol [4].

The association between non-disclosure of HIV serostatus to sex partners and self-reporting of defaulting on ARVs, not picking up ARVs from the clinic and peripartum viral load non-suppression as reported in this study suggest that non-disclosure of HIV serostatus could impact the outcomes of PMTCT. Of critical importance is the finding of the increased odd of high viral load at delivery among women who did not disclose their status to their partners. This has serious implications for the transmission of HIV from mother-to-child and between sex partners in the study setting. This finding is similar to Trinh et al [16] and Jasseron et al [26] in Kenya and France, respectively. The plausible explanation for this finding is that non-disclosure of serostatus could hinder women from picking up of ARVs and adhering to use of medications. A previous study reported the link between non-disclosure of serostatus and poor adherence of ARVs [10].

Reasons for non-disclosure vary by individual and their partners, however, fear of rejection, violent abuse, having to take responsibility for transmission risk, and not disclosing at the initiation of the relationship are the reasons for non-disclosure as detailed in the findings of this study. This finding is similar to extant literature [4, 5, 18–20, 24, 30] but differs by introducing non-disclosure at the start of the relationship to the discourse. South Africa was the first country in Africa to approve the use of pre-exposure prophylaxis (PrEP) for prevention [40]; however, it is too early to assess to what extent the availability of PrEP would provide an incentive for partner testing or disclosure. Nonetheless, PrEP would potentially prevent horizontal transmission in serodiscordant relationships.

The limitations of this study cannot be ignored. Social desirability bias from self-reporting of disclosure measures cannot be excluded. Nevertheless, the results are reported and admitted non-disclosure and this likely represents a minimum percentage of partners and infants at risk for transmission. The cross-sectional design of the study did not allow for causal association to be drawn. The large sample size and the multi-centre nature of the study support the degree of representativeness of the study sample, which gives credence to the findings. Future studies from this cohort will allow for monitoring of the women and their partners.

## Conclusion

Non-disclosure of HIV serostatus is a public health concern with serious implications for the prevention of mother-to-child transmission programme; and also, reduction in new infections from horizontal transmission at the population level. Strategic efforts toward ending the epidemic of HIV and AIDS in South Africa should address the sociocultural and behavioural determinants of non-disclosure. Also, the link between self-reporting of non-adherence to ARVs, not picking up of ARVs from the clinics, and peripartum virological failure in individuals who were yet to disclose their serostatus to their partners could potentially inform core patient education at diagnosis of HIV and during counseling sessions. The findings of this study suggest that non-disclosure of HIV serostatus to sex partners could impede the success of PMTCT. Couple testing and counseling could be a long-term goal at improving HIV disclosure and partner involvement.
